# The economic cost of outpatient primary care of adults with multimorbidity (HIV, diabetes, and hypertension) in rural South Africa

**DOI:** 10.1093/heapol/czag016

**Published:** 2026-02-10

**Authors:** Celeste Claire Holden, Winfrida Mdewa, Theophilous Mathema, Chodziwadziwa Whiteson Kabudula, Kayode Adetunji, Roy Zent, Susan Goldstein, Kerry Glover, Scott Hazelhurst, Michael Klipin, Stephen Tollman, Francesc Xavier Gómez-Olivé, Evelyn Thsehla

**Affiliations:** SAMRC/Wits Centre for Decision Science and Health Economics (PRICELESS SA), Wits Health Consortium, University of the Witwatersrand, 27 St Andrews Rd, Parktown, Johannesburg 2193, South Africa; SAMRC/Wits Centre for Decision Science and Health Economics (PRICELESS SA), Wits Health Consortium, University of the Witwatersrand, 27 St Andrews Rd, Parktown, Johannesburg 2193, South Africa; Sydney Brenner Institute for Molecular Bioscience (SBIMB), University of the Witwatersrand, 9 Jubilee Rd, Parktown, Johannesburg 2193, South Africa; SAMRC/Wits Rural Public Health and Health Transitions Research Unit, School of Public Health, Faculty of Health Sciences, University of the Witwatersrand, 27 St Andrews Rd, Parktown, Johannesburg 2193, South Africa; Sydney Brenner Institute for Molecular Bioscience (SBIMB), University of the Witwatersrand, 9 Jubilee Rd, Parktown, Johannesburg 2193, South Africa; Division of Nephrology, Department of Medicine, Vanderbilt University, 1161 21st Ave S, Nashville, TN 37232, United States; SAMRC/Wits Centre for Decision Science and Health Economics (PRICELESS SA), Wits Health Consortium, University of the Witwatersrand, 27 St Andrews Rd, Parktown, Johannesburg 2193, South Africa; Sydney Brenner Institute for Molecular Bioscience (SBIMB), University of the Witwatersrand, 9 Jubilee Rd, Parktown, Johannesburg 2193, South Africa; SAMRC/Wits Rural Public Health and Health Transitions Research Unit, School of Public Health, Faculty of Health Sciences, University of the Witwatersrand, 27 St Andrews Rd, Parktown, Johannesburg 2193, South Africa; Sydney Brenner Institute for Molecular Bioscience (SBIMB), University of the Witwatersrand, 9 Jubilee Rd, Parktown, Johannesburg 2193, South Africa; School of Electrical & Information Engineering, University of the Witwatersrand, 1 Jan Smuts Ave, Johannesburg 2000, South Africa; Department of Surgery, Faculty of Health Sciences, University of the Witwatersrand, 7 York Rd, Johannesburg 2193, South Africa; WitsBits, Health Sciences Biomedical Informatics, Wits Health Consortium, 31 Princess of Wales Terrace, Parktown, Johannesburg 2193, South Africa; SAMRC/Wits Rural Public Health and Health Transitions Research Unit, School of Public Health, Faculty of Health Sciences, University of the Witwatersrand, 27 St Andrews Rd, Parktown, Johannesburg 2193, South Africa; SAMRC/Wits Rural Public Health and Health Transitions Research Unit, School of Public Health, Faculty of Health Sciences, University of the Witwatersrand, 27 St Andrews Rd, Parktown, Johannesburg 2193, South Africa; SAMRC/Wits Centre for Decision Science and Health Economics (PRICELESS SA), Wits Health Consortium, University of the Witwatersrand, 27 St Andrews Rd, Parktown, Johannesburg 2193, South Africa

**Keywords:** multimorbidity, South Africa, cost of illness, HIV, diabetes, hypertension, NCDs

## Abstract

Sub-Saharan Africa (SSA) is experiencing an epidemiological transition where non-communicable diseases are becoming the leading cause of disability and mortality alongside infectious diseases such as HIV/AIDS. Multimorbidity, the coexistence of two or more long-term conditions, is increasing in SSA. However, the cost of managing multimorbidity is largely unknown. This study aimed to estimate the economic cost of public outpatient primary care for adults with multimorbidity (HIV, hypertension, and/or diabetes, and their associated conditions: cardiovascular disease, and TB) in rural South Africa. This study used a cross-sectional, retrospective cost-of-illness approach to estimate the direct and indirect costs of multimorbidity management in Bushbuckridge, Mpumalanga, in 2022. Data were synthesized from patient-level data from eight public primary healthcare facilities within the Agincourt study site—a rapidly transitioning rural South African setting. Additionally, government reports and an existing study on transport costs and productivity losses conducted within the Agincourt study site were used to estimate the costs of managing patients in the primary care facilities. Results showed that patients with multimorbidity had higher average economic costs per patient compared to those with single conditions. Overall, patients with multimorbidity increase costs above the baseline of a patient with a single condition (R4 900/annum) by between 42% and 83%. Patients with multimorbidity also incur slightly higher costs associated with accessing primary care services compared to those with a single condition. However, our model shows that the additive cost of managing multiple conditions in separate consultations is higher than managing all conditions in one visit. This shows that managing patients within an integrated care model seems to have a cost-limiting effect. However, treatment guidelines for managing multimorbidity in South Africa should be developed to ensure standardized care.

Key messages

- The prevalence of NCDs such as diabetes and hypertension is rapidly increasing in South Africa. These conditions, when combined with the high infectious disease burden, such as HIV, prove a challenge to healthcare system in SA and other LMICs, particularly in SSA.
- Multimorbidity increases direct healthcare costs when compared with those with just a single condition due to the additional medications, laboratory tests, and visits to healthcare facilities.
- Indirect costs, particularly productivity losses, represent a significant portion of the overall economic burden of all conditions but more so for those with multimorbidity.
- Resources should be used to devise standard treatment guidelines for multimorbidity in SA as a starting point.
- Integrated care models seem to have a cost-limiting effect. However, the quality of integrated care in this study was not investigated.

## Background

Multimorbidity poses one of the greatest emerging challenges for health care systems worldwide. In 2021, more than half of the adult population above 60 years of age had multimorbid conditions (Rahman Chowdhury et al. 2023, Tran 2024). Multimorbidity is commonly defined in the literature as the coexistence of two or more chronic conditions (Soley-Bori et al. 2021, Micklesfield et al. 2023, Rahman Chowdhury et al. 2023). The high prevalence of multimorbidity significantly adds to the overall burden of illness, affecting society through increased disability, reduced functionality, and higher risks of mortality (Rahman Chowdhury et al. 2023, Tran 2024, Tran et al. 2024).

Sub-Saharan Africa (SSA) is undergoing a social and epidemiological shift where non-communicable diseases (NCDs) are overtaking infectious disease as the leading cause of disability and mortality (Nyirenda 2016, Mudie et al. 2019, Tamuhla et al. 2021). The prevalence of multiple conditions is estimated at 47.2% in women and 35% in men in Ghana, Kenya, Burkina Faso, and South Africa (Micklesfield et al. 2023). The increased burden of NCDs alongside infectious diseases has been linked to rapid urbanization, increased life expectancy, and the globalization of the food industry (Mudie et al. 2019, Onagbiye et al. 2020, Tamuhla et al. 2021, Dzomba et al. 2023, Micklesfield et al. 2023). In South Africa, this epidemiologic transition is evident with the country reporting one of the highest levels of overweight and obesity in the world (Manafe et al. 2022, World Obesity Federation 2024), a major contributing factor to hypertension, cardiovascular disease, and diabetes mellitus type 2 (DMT2). The rise of NCDs alongside an already high burden of infectious diseases has increased the prevalence of multimorbidity placing an increased demand on an already overburdened and under-resourced health system (Lalkhen and Mash 2015, Gouda et al. 2019, Dixon et al. 2024). Additionally, there is research to show that multimorbidity in South Africa is consistently significantly associated with income, with the increased burden placed on the poorer populations (Alaba and Chola 2013). Prevalence studies of multimorbidity in South Africa state that multimorbidity is ‘the norm’ in South Africa, especially among older adults (Roomaney et al. 2021, Roomaney et al. 2023). However, the economic burden associated with multimorbidity in South Africa, particularly including NCDs, is not yet understood.

Literature on the cost of multimorbidity often focuses on specific combinations of diseases which can be dyads (combinations of two co-existing conditions) and triads (combinations of three or more co-existing conditions) (Dixon et al. 2024). A systematic review of health care utilization and costs of older people with multimorbidity found that this population had consistently more doctor visits, experienced more hospital stays, and inpatient bed days, and consumed more pharmaceuticals than other patient populations (Lehnert et al. 2011). A more recent systematic review found that resource utilization and costs are dependent on the combination of diseases and the age of the patient population (Soley-Bori et al. 2021), for example, depression has been found to be a cost-increasing comorbidity while hypertension ends to be cost-limiting (which means that an additional diagnosis of hypertension does not significantly increase the cost of accessing healthcare) (Brilleman et al. 2013, Soley-Bori et al. 2021). However, these findings are mainly (95%) from high-income countries with only 5% conducted in middle-income countries and none recorded in low-income countries (Tran et al. 2022, 2024).

Studies that estimate the cost of multimorbidity in South Africa are scarce. Mudzengi et al. (2017) estimated the cost of multimorbidity focusing on HIV and TB in South Africa (Mudzengi et al. 2017). This study found that people with both TB and HIV are at higher risk of catastrophic healthcare expenditure, defined in this study as ≥10% of individual income (Mudzengi et al. 2017). Additionally, this study found that the integration of services reduced the number of standalone TB and HIV visits to the health facility, thus reducing the burden on healthcare workers (HCWs) and direct non-medical patient costs such as travel (Mudzengi et al. 2017).

Multimorbidity studies have been largely overlooked in South Africa due to limitations related to routine health information systems as well as the current focus on single diseases (Roomaney et al. 2023). Based on what we currently know about the increase in NCD prevalence and the trends identified in other countries, multimorbidity is likely to be a signiﬁcant contributor to ill health in South Africa (Roomaney et al. 2023). The aim of this study was to estimate the economic cost of public outpatient primary care for adults with multimorbidity (HIV, hypertension, and/or diabetes, and their associated conditions, cardiovascular disease, and TB) in rural South Africa.

The three primary conditions were included because of their high prevalence and burden in South Africa and specifically in the Mpumalanga province. Hypertension affects nearly half of the South African population (44%) with nearly half of these hypertension cases remaining undiagnosed and only 46% of diagnosed cases receiving treatment (Department of Health 2019, World Health Organization 2023). Furthermore, diabetes prevalence and mortality are also on the rise in South Africa, affecting 10.1% of the population (International Diabetes Federation 2021). In 2022, diabetes was the second leading cause of death and the fifth and sixth leading cause of disability-adjusted life year losses in Mpumalanga and SA, respectively (Institute for Health Metrics and Evaluation 2023, The Institute for Health Metrics and Evaluation 2019). Similar to hypertension, nearly half of diabetes cases remain undiagnosed (International Diabetes Federation 2021). The high level of undiagnosed cases, low level of treatment rates, and high prevalence of these NCDs make them crucial to investigate (Roomaney et al. 2021, Roomaney et al. 2023).

HIV was also included because South Africa has one of the highest HIV prevalence in the world. In 2022, 13.9% of the population were HIV-positive. (Massyn et al. 2020, Statistics South Africa (Stats SA) 2022b). Scaling up antiretroviral therapy (ART) to reach the target of 95% treatment coverage over the next 20 years is estimated to cost R117bn, including R58bn for additional ART and healthcare worker costs (UNAIDS 2021). To our knowledge, there have been no studies focused on the cost of these NCDs alongside an infectious disease like HIV in South Africa. This study aims to meet this gap.

This study takes place in Agincourt, located in the Bushbuckridge district of rural north-eastern South Africa. The South African National Department of Health (NDoH) developed an Integrated Chronic Disease Management (ICDM) model which was piloted in 2011–2013 and then implemented into the ‘ideal clinic’ framework from 2014 throughout the country (National Department of Health (NDoH) 2014). The ICDM focuses on prevention and self-management of both communicable diseases like HIV and tuberculosis, and NCDs such as hypertension, diabetes, asthma, and common mental health illnesses (National Department of Health (NDoH) 2014). The chronic clinics in Mpumalanga, including the clinics in the Agincourt study site, fall under the ideal clinics framework and are thus guided by the ICDM. However, the ICDM is not utilized directly by healthcare workers but is rather utilized by clinics’ operational managers (OMs) who use the ICDM to ensure compliance, improve staff values and attitudes, waiting times, cleanliness, patient safety and security, infection prevention and control, and the availability of medicines and supplies (National Department of Health (NDoH) 2014).

The Agincourt study site provides a compelling setting for estimating the cost of multimorbidity in rural South Africa due to its well-defined rural population and the rich longitudinal data available on this population (MRC/Wits Rural Public Health and Health Transitions Unit (Agincourt); and (SAPRIN) 2025). This study is novel as its focus is on patients with NCDs and communicable diseases due to their continual rise in prevalence in South Africa.

## Methods

### Study design

This is a cross-sectional, retrospective, cost-of-illness (COI) study using a prevalence approach. This study used a worksheet-based COI model to synthesize data from multiple sources. The COI model adopted a ‘bottom-up’ (person-based) approach to estimate the direct and indirect cost of diagnosis and management of multimorbidity in public primary healthcare (PHC) facilities (Larg and Moss 2011).

### Study setting

The study settling is known as the ‘Agincourt study site’, which resides in the Agincourt-Bushbuckridge sub-district in the Mpumalanga province, Northeast South Africa. The Agincourt study site is categorized by a burden of disease ‘driven by a trio of conditions: HIV, hypertension, and obesity’ (Dzomba et al. 2023). As documented by the Agincourt health and socio-demographic surveillance system (HDSS), the Agincourt-Bushbuckridge sub-district comprises of about 116 000 people residing in 21 000 households in 31 villages (MRC/Wits Rural Public Health and Health Transitions Unit (Agincourt) 2025). Our focus was on the network of eight PHC clinics within the Agincourt study site. Each clinic has one or more data capturers (a contribution of the Agincourt Research Unit) that enters data from patient files to the Clinic Link system daily. All PHC facilities endeavour to offer integrated care for the conditions included in this study through the ‘chronic clinics’, which are a dedicated service provided by each PHC facility that caters for the screening and management of common chronic conditions (HIV/AIDS, hypertension and diabetes, and more). In most cases, patients with chronic illnesses have all their conditions addressed in one appointment. All eight clinics in the Agincourt study site fall within the ideal clinics framework which is guided by the ICDM framework. The direct healthcare costs of obtaining primary healthcare in the Agincourt study site should be representative of other rural areas in South Africa as these are defined at a national level. However, if looking at another specific rural area of South Africa, certain parameters would need to be changed accordingly such as disease prevalence and employment rate as these may differ between regions of South Africa.

### Study population

The study population comprised adults (aged 18 years or older) with one or more of the following conditions: HIV/AIDS, hypertension and diabetes who attended at least one of the eight PHC clinics in 2022. The single condition groups include patients with only a single diagnosis and no other conditions. The patients with multimorbidity were grouped by their conditions and have no additional diagnoses other than those listed.

### Study parameters and data sources

This study uses Agincourt HDSS-Clinic-Hospital link data (‘Clink Link’ data) collected from facilities in Agincourt by data staff of the SAMRC/Wits Rural Public Health and Health Transitions Unit (Agincourt) in 2022 (1 January 2022–31 December 2022). The Clinic Link dataset is a longitudinal repository that records patient information from eight PHC facilities, namely: Agincourt community healthcare centre (CHC), Belfast Clinic, Cunningmoore Clinic, Justicia Clinic, Kildare Clinic, Lillydale Clinic, Thulamahashe CHC, and Xanthia Clinic. The dataset includes a unique patient identifier, patient diagnoses, visit dates, medication prescribed at each visit, and a second dataset which records patient laboratory tests at each visit in 2022. Supplementary Appendix S1 illustrates the format of the data, and the variables used in this study (Supplementary Appendix S1). The Agincourt study site is a member of the South African Population Research Infrastructure Network (SAPRIN) (South African Population Research Infrastructure Network (SAPRIN) 2025).

Data for the cost of illness (COI) model were collected on key parameters within the following data categories: population estimates, epidemiology estimates, treatment coverage, direct medical costs, and indirect medical costs. Data sources for the study parameters are outlined in Table 1. The Clinic Link data informed non-cost parameters such as the disease prevalence in Agincourt and the number of clinic visits for each condition group in 2022.

**Table 1:** Data categories, model parameters, and data sources used to inform the cost.

Data category	Parameter	Sources
Population estimates	Adult Population utilising the public healthcare sector	Agincourt patient-level data (Clinic Link) Statistics South Africa (Stats SA)
Epidemiology estimates	Prevalence Disease burden	Agincourt patient-level data (Clinic Link) District Health Barometer (DHB) 2019/20 Statistics South Africa (Stats SA) Literature
Treatment coverage	Screened, diagnosed, treated, and controlled cases	Agincourt patient-level data (Clinic Link) Literature
Direct medical costs	Facility fees Medications Examinations and laboratory investigations Healthcare worker wages	Uniform Patient Fee Schedule Department of Health (DoH) tenders (2021–2025) Medicine Price Registry 2024 South African Standard Treatment Guidelines 2020 South African National Health Laboratory Service (NHLS) 2018 NDoH HCW vacancy advertisements Journal of Endocrinology, Metabolism and Diabetes of South Africa (JEMSDA) Guidelines (2017) Road Accident Fund Medical Tariffs (2023) Agincourt patient-level data (Clinic Link) Primary data collection in Agincourt through an adjacent study
Indirect medical costs	Transport costs Productivity losses	Primary data collection in Agincourt through an adjacent sub-study Various government reports

### Identification of resources

Table 1 outlines a list of all the sources used to get information required to estimate the cost of managing each condition group at a primary level in Agincourt. First, the Clinic Link data provided patient-level data on the resources used to manage patients with HIV/AIDS, diabetes, and hypertension, including the number of visits each patient made to the clinic per patient, the medication prescribed per visit, and the laboratory tests requested at each clinic visit. We determined which medications to include and the dose of medication by consulting the standard treatment guidelines (STGs). The medications included in the model are outlined in Supplementary Appendix S8 and S9 and only include those associated with HIV/AIDS, hypertension and diabetes and their associated conditions: cardiovascular related conditions and TB. Importantly, the patients included in this study only had one or more of the primary conditions as recorded in the diagnosis section of the Clinic Link dataset (HIV/AIDS, HT, and/or DM) and did not have any other diagnoses even if they were screened for and/or managed for additional conditions. Supplementary Appendix S8 outlines how missing values for each medication were handled, and Supplementary Appendix S9 shows which variables were grouped together (due to the variables in the Clinic Link dataset being string variables and so spelling and formatting errors needed to be addressed).

The identification of laboratory tests was informed by the Clinic Link dataset and then, as with medications, we used the STGs to ensure we included only the disease-specific tests in the model. Supplementary Appendix S10 outlines the laboratory tests considered in this study as taken from the Clinic Link dataset, and their corresponding South African National Health Laboratory Service (NHLS) test name (National Health Laboratory Service 2018), as the names often differed between the two. There were no data on investigations and consumables in the Clinic Link dataset, and so identification of resource use for additional investigations and consumables was based on the STGs (The National Department of Health 2020) and additional literature review (Mudzengi et al. 2017, Erzse et al. 2019, International Diabetes Federation 2021, Kohli-Lynch et al. 2022) and further validated by the operations managers at three of the eight clinics. *Investigations* included blood pressure (BP) monitoring and electrocardiograms (ECGs), while *consumables* included syringes and needles for insulin users, lancets, glucose strips, and providing insulin-dependent diabetic patients with battery-powered glucometers for home use.

Information on the HCWs who work at each of the eight facilities and the frequency at which each HCW attends each clinic was obtained from data typists based in Agincourt clinics (see Supplementary Appendix S6). These included nurses, OMs, community healthcare workers (CHWs), medical officers (MOs), specialist physicians, pharmacists, pharmacist assistants, dietitians, psychologists, HIV counsellors, optometrists, audiologists, and rehabilitation therapists, including physiotherapists, occupational therapists, and speech therapists (Supplementary Appendix S6).

### Quantification of resources

Resources were quantified by analysing the 2022 Clinic Link data using STATA 16.0 statistical software (STATA 2025). A descriptive analysis allowed us to determine the total number of visits, units of medication prescribed per visit, and the type and number of laboratory tests ordered per visit per patient. The dataset did not contain information on the dose of medication prescribed for each patient, so doses recommended in the STGs were used (The National Department of Health 2020). We quantified the frequency at which each healthcare professional worked across the eight facilities based on information received from the Agincourt team (Supplementary Appendix S6). Furthermore, a literature review was conducted to inform the extent each patient group requires the services of each type of healthcare professional. Community healthcare workers were presumed to see each patient at every visit, even if just to take their vitals upon entering the clinic as observed in three of the eight clinics. Another assumption made is that if a patient was prescribed medication at a visit, the patient must have been seen by a nurse and required the services of a pharmacist and pharmacy assistant as well as the Operations Manager (even though these are not direct interactions). Supplementary Appendix S11 outlines the literature and the assumptions made to inform the proportion of HCWs attending to each patient group so that we were able to attribute part of their cost to each specific group.

### Estimation of direct medical costs

The total and per patient healthcare cost for each condition group was estimated using the bottom-up gross costing approach (Hendriks et al. 2014), by identifying services used, measuring the units (in the case of medicines, laboratory tests, consumables, and additional investigations), and assigning a monetary value using appropriate tariffs and unit prices in the public sector.

The unit costs to determine the direct medical costs were largely informed by publicly available government reports and documents including government tenders for medication and the Medicine Price Registry (National Department of Health 2020, 2022, 2023, Medical Price Registry 2024). The unit costs for laboratory tests were taken from the most recent publicly available price list published by the NHLS (National Health Laboratory Service 2018). HCW annual salaries were taken from government vacancy advertisements (The National Department of Health 2023). Facility fees were outlined in the Uniform Patient Fee Schedule (The South African National Department of Health 2023). The salaries of CHWs were provided to us by the Agincourt team. Cost data for additional investigations and consumables were informed by literature review including costs outlined by the 2023 Road Accident Fund Medical tariff list (Department for transport, 2023) and the Society for Endocrinology, Metabolism and Diabetes of South Africa (SEMSDA) 2017 guidelines (Dave and Coetzee 2019).

Medicines and laboratory tests were estimated using the Clinic Link data which gave the exact amounts of units prescribed for each patient, per visit. Medicine costs were calculated by multiplying the unit costs of each medication by the number of units of that medication prescribed in 2022, per condition group. Similarly, laboratory test costs were calculated by multiplying the unit cost for each laboratory test by the number of laboratory tests done per condition group in 2022.

The number of investigations done (such as BP monitoring and ECGs) and the consumables used or given out to patients (such as glucose strips, syringes, needles, and glucometers) were not available in the Clinic Link dataset. Thus, assumptions were made based on literature (see ‘identification of resources’) and consultation with the OMs at the clinics. For example, we were informed that every patient who enters the clinic gets their blood pressure taken regardless of diagnosis and so in our model all patients were charged a fee for BP monitoring. Another assumption confirmed by the OM was that glucose strips, syringes, needles, and glucometers are only given to insulin dependent patients and so only these patients are allocated these costs. We then calculated the cost by multiplying the unit cost for each investigation and/or consumable by the estimated number of investigations conducted or consumables used for each condition group.

Total HCWs costs were calculated by multiplying a portion of each HCW’s annual salary in 2022 by the total number of HCWs who work across the eight facilities, multiplied further by the frequency by which they work in the PHC facilities (see Supplementary Appendix S6). For example, in the case of doctor’s fees, we used the Medical Officer Grade 1 annual salary in 2022 (ZAR 857 027, 84), which is known as full-time equivalent (FTE). We then multiplied by this annual salary 1.4 to account for the frequency by which the doctors work at the facilities. In this case, there are six doctors who work across the eight facilities. Five of the six doctors visit each clinic one day per week resulting in 20% (or 0.2) of each doctor’s annual salary attributable to offering PHC services across the eight facilities (0.2 × 5 = 1.0 FTE) and one doctor who goes into a clinic twice a week (0.4 × 1 = 0.4 FTE) resulting in a 1.4 FTE. We then multiplied this by the proportions of patients in each condition group who may require the expertise of a doctor to get the cost per condition group. This was based on literature and various assumptions made, validated by the OMs who work in three of the eight clinics (see Supplementary Appendix S10).

### Estimation of indirect costs and direct non-medical costs

Indirect costs were defined in this study as patient productivity losses as a result of accessing PHC care. *Direct non-medical* costs included patient travel costs associated with accessing PHC care. The sources for unit costs are outlined in Table 1. To calculate productivity losses, we multiplied the median daily wage of ZAR 170,00 (10,59 USD) by the number of employed patients (17%) visiting the clinic in each condition group (see Table 2). Similar to productivity losses, the main source of information for direct non-medical costs (transport costs) were gleaned from data gathered from the Agincourt study site (see Supplementary Appendix S2 and S4). The average cost for a round-trip to the clinic for one visit was ZAR 46,00 (2.87 USD) with public taxis being the most common form of transport (Supplementary Appendix S2 and S4). This transport cost was multiplied by the number of visits in each condition group to get the total cost of transport per condition group.

**Table 2:** Descriptive characteristics of the adult population accessing primary healthcare services at the eight clinics within the Agincourt study site, 2022.

		Total number of patients	Proportion of the total (%)	Total number of visits	Proportion of the total (%)
Total sample		20 025	100.0	105 815	100.0
Single conditions	HIV-only	12 844	64.1	66 648	63.0
	DM-only	165	0.8	841	0.8
	HT-only	3785	18.9	19 548	18.5
	Total single conditions	16 794	83.8	87 037	82.3
Multiple conditions	DM & HT	715	3.6	4416	4.2
	DM & HIV	81	0.4	526	0.5
	HT & HIV	2274	11.4	12 810	12.1
	HIV & DM & HT	161	0.8	1026	1.0
	Total multiple conditions	3231	16.1	18 778	17.7

### Estimation of catastrophic expenditure

The principle of catastrophic costs is rooted in identifying when patients and their households involuntarily reduce expenditure on basic household needs such as food, clothing, and education in order to pay for healthcare. All direct medical costs were undertaken by the health system as healthcare in Agincourt is free at the point of care. Thus, we only considered the direct non-medical costs: transport costs and indirect costs including productivity losses of patients. According to the World Health Organization, costs are defined as catastrophic when total costs incurred (direct and indirect costs combined) exceed a given threshold of household income (World Health Organization 2024). However, in the absence of reliable household income data, we used a similar approach to the COI study done in SA on TB and HIV (Mudzengi et al. 2017) and compared the expenditure to the median monthly income obtained from our sub-study which was R2 900 (181 USD) (Supplementary Appendix S4). We used a threshold of 10% of individual income, which has been a widely used benchmark for catastrophic costs in many patient costing studies due to the challenges of measuring household income (Xu et al. 2003, Tanimura et al. 2014, Wingfield et al. 2014, Mudzengi et al. 2017).

### Cost adjustment

The study results are reported using the South African Rand (ZAR) and converted into the United States Dollar (USD) using the 2022 mid-year exchange rate of 1USD = 16,05 ZAR (OANDA no date). Cost data before and after 2022 were adjusted using consumer price index (CPI) midyear values (Statistics South Africa (Stats SA) 2022a, 2024). In cases where the tender price for a medication was from a 2023 or 2024 document, CPI values were used to adjust these prices into 2022 units.

### Scenario analysis

We explored four different scenarios to assess their effect on the economic costs of multimorbidity over 20 years using 2022 as our baseline. The parameters in the scenario analysis were chosen as they were agreed to be the most susceptible to change over time and thus to have the biggest potential effect on the cost of multimorbidity in future. The first scenario was an increase in the prevalence of multimorbidity (%) which we increased by 1% per year. The second scenario was an increase in the frequency of doctors working at PHC clinics which we increased by 1.0 per year (which can be understood as increased time that doctors spend at the PHC clinics resulting in an increase in the portion of their salaries being attributed to primary care). The third scenario was the annual CHW wage which we increased by 2% per annum. The fourth scenario was an increase in the employment rate in the Agincourt study site (increased by 1% per annum).

## Results

### Descriptive characteristics

In 2022, a total of 20 025 patients with one of the following conditions: HIV, diabetes and/or hypertension accessed one of the eight public PHC clinics in the Agincourt study site (see Table 2). A total of 105 815 clinic visits with an average of 5.28 visits per patient per annum were recorded (Tables 2, 3). Approximately 16.1% of the study population had multimorbidity as defined by our study (two or more of the following conditions: HIV, diabetes, and/or hypertension) with the combination of HIV and hypertension being the most common type of multimorbidity (11.4%) (Table 2). The average number of visits per annum for patients with any single conditions was 5.15, which is lower than the average number of visits per annum for patients with multiple conditions at 5.81 (Table 2). Patients with both diabetes and HIV had the highest average number of annual visits with at 6.49, followed closely by patients with all three conditions (HIV, DM, and HT) with an average of 6.37 visits (Table 3). The most prevalent condition in the sample was ‘HIV-only’ making up 64.1% of the sample with the ‘diabetes and HIV’ group having the lowest prevalence, making up only 0.4% of the sample (Table 2).

**Table 3:** Clinic visits, direct medical costs, direct non-medical costs and indirect costs per patient per annum by condition type, Agincourt study site, 2022.

Conditions		Annual number of clinic visits (mean)	Direct medical costs per patient per annum (ZAR/USD)					Total costs per patient per annum (ZAR/USD)			
			HCW fees	Medication	Laboratory tests	Consumables and investigations	Facility fees	Total direct medical costs	Total direct nonmedical costs	Total Indirect costs	Total Economic costs
Single conditions	HIV-only	5.19	2608/ 162	786/ 49	304/ 18.9	707/ 44	586/ 37	4987/ 311	239/ 14.9	128/ 8.0	5353/ 334
	DM-only	5.10	2567/ 160	1336/ 83	20/ 1,3	778/ 49	576/ 36	5277/ 329	234/ 14.6	126/ 7.8	5637/ 351
	HT-only	5.16	2567/ 160	562/ 35	18/ 1.1	706/ 44	583/ 36	4435/ 276	238/ 14.8	127/ 7.9	4800/ 299
	Total single conditions (mean)	5.15	2580/ 161	893/ 56	114/ 7.1	730/ 136	582/ 36	4900/ 305	237/ 14.8	127/ 7.9	5263/ 328
Multiple conditions	DM & HT	6.18	2567/ 160	2118/ 132	12/ 0.7	781/ 49	697/ 43	6175/ 385	284/ 17.7	152/ 9.5	6611/ 412
	DM & HIV	6.49	5184/ 323	1986/ 124	211/ 13.2	832/ 52	733/ 46	8947/ 557	299/ 18.6	160/ 10.0	9406/ 586
	HT & HIV	5.63	2567/ 160	1184/ 74	340/ 21.2	728/ 45	636/ 40	5455/ 340	256/ 16.1	139/ 8.7	5853/ 365
	HIV & DM & HT	6.37	2608/ 162	2588/ 161	283/ 17.6	789/ 49	720/ 45	6988/ 435	293/ 18.3	157/ 9.8	7438/ 463
	Total multiple conditions (mean)	5.81	2567/ 160	1969/ 123	211/ 13.2	783/ 49	697/ 43	6891/ 429	284/ 17.7	152/ 9.5	7327/ 457
Total sample		5.28	3018/ 188	858/ 53	241/ 15.0	713/ 44	597/ 37	5013/ 312	243/ 15.1	130/ 8.1	5386/ 336

### Direct medical costs

**Figure 1:**
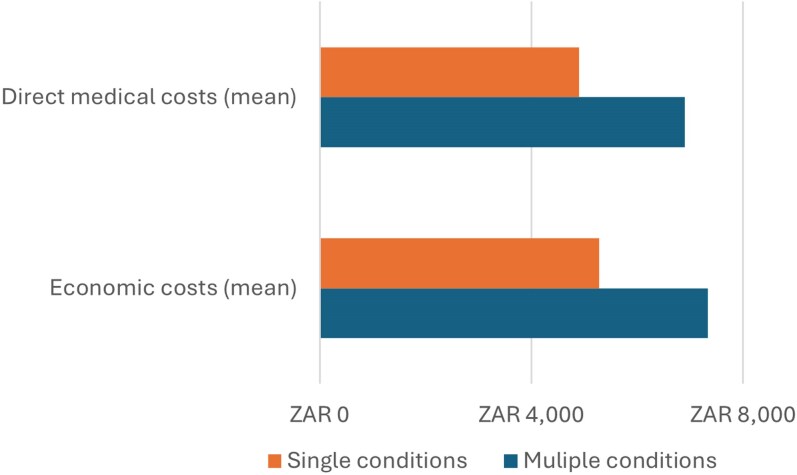
Average annual direct medical and economic costs, Agincourt study site, 2022: single conditions compared to multimorbidity.

**Figure 2:**
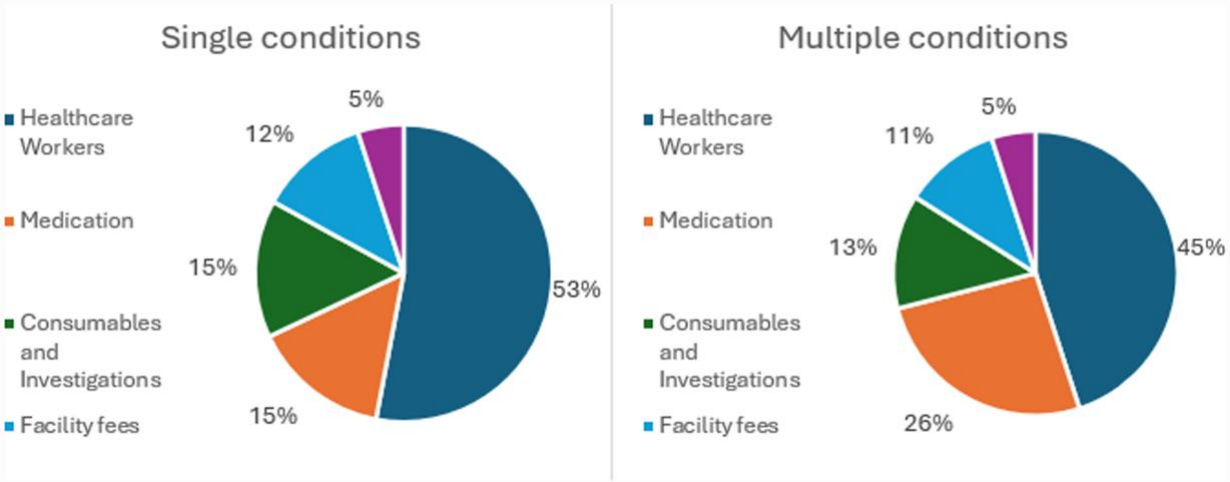
Annual direct medical costs associated with accessing public primary healthcare services, Agincourt study site, 2022.

The annual direct medical costs by condition groups are outlined in Tables 3 and 4, with Table 3 presenting the costs per patient in each condition group and Table 4 presenting the total annual costs by condition group. Patients with ‘HIV and diabetes’ had the highest direct medical costs per patient at ZAR 8947 (557 USD) per annum while patients with ‘hypertension-only’ had the lowest overall cost per patient at ZAR 4435 (276 USD) per annum (Table 3). Figure 1 also illustrates the direct medical costs for those with multimorbidity are higher than for patients with a single condition. The average direct medical costs for patients with a single condition is ZAR 4900 (305 USD) versus ZAR 6981 (429 USD) for the multimorbidity patients (Table 3). Table 4 focuses on the total annual direct medical costs of managing patients, the ‘single condition’ patient groups contribute the highest to the annual direct medical costs overall due to their high prevalence, with the ‘HIV-only’ group contributing the largest proportion of direct medical costs (ZAR 64 049 513/3 990 624 USD) per annum reflecting the high prevalence of HIV in this patient population (Tables 2 and 4). We then looked at the breakdown of the various cost categories which make up the total direct medical costs (Fig. 2), and we observed that HCW fees made up the highest proportion of direct medical costs for both groups, followed by medication, with the lowest cost contributor being laboratory tests. Figure 2 also shows that healthcare worker costs constitute a higher proportion of the direct medical costs for those with single conditions, whereas medication costs constitute a higher proportion of direct medical costs for patients with multiple conditions.

**Table 4:** Total annual costs by condition, Agincourt study site, 2022.

Conditions		Direct medical costs	Direct non-medical costs: travel costs	Indirect costs: patient productivity losses	Total economic costs
		Total per annum (ZAR/USD)			
Single conditions	HIV-only	64 049 513/ 3 990 624	3 065 808/ 191 016	1 642 873/ 102 360	68 758 194/ 4 284 000
	DM-only	870 629/ 54 245	38 686/ 2410	20 731/ 1292	930 046/ 57 947
	HT-only	16 788 175/ 1 045 992	899 208/ 56 025	481 858/ 30 022	18 169 241/ 1 132 040
	Total single conditions	81 708 317/ 5 090 861	4 003 702/ 249 452	2 145 462/ 133 674	87 857 481/ 5 473 986
Multiple conditions	DM & HT	4 415 094/ 275 084	203 136/ 12 656	108 854/ 6782	4 727 084/ 294 522
	DM & HIV	724 703/ 45 153	24 196/ 1508	12 966/ 808	761 865/ 47 468
	HT & HIV	12 404 540/ 772 869	589 260/ 36 714	315 767/ 19 674	13 309 567/ 829 256
	HIV & DM & HT	1 125 004/ 70 094	47 196/ 2941	25 291/ 1576	1 197 491/ 74 610
	Total multiple conditions	18 669 340/ 1 163 199	863 788/ 53 819	462 878/ 28 840	19 996 006 1 245 857
Total sample		100 377 657/ 6 254 060	4 867 490/ 303 270	2 608 340/ 162 513	107 853 487/ 6 719 843

### Economic cost

Table 3 outlines the per patient annual economic costs by condition group with Table 4 giving the annual total economic cost of each condition group. In Table 3, multimorbidity patients are seen to have higher costs per patient for all cost categories. The economic costs for patients with multimorbidity are higher per patient (ZAR 7327/457 USD) than those with single conditions (ZAR 5263/328 USD) (Fig. 1; Table 3). Patients with ‘HIV and diabetes’ have the highest economic cost per patient per annum at ZAR 9406 (586 USD), which is almost double the cost of the ‘HT-only’ group, which costs ZAR 4800 (299 USD) per patient per annum. Table 4 shows that the total annual costs are higher for patients with single diseases (ZAR 81 708 317/5 090 861 USD) almost four times higher than the total annual costs to manage multimorbidity patients (ZAR 18 669 340/1 163 199 USD). This is due to the high patient numbers in the ‘single condition’, especially the ‘HIV-only’ group, which makes up 64.1% of the total sample (Table 2).

### Catastrophic health expenditure

Table 5 shows the direct non-medical costs (transport costs) and indirect costs (patient productivity losses) associated with each condition category. Individuals with multiple conditions had higher annual average costs in both categories with the ‘diabetes and HIV’ group experiencing the highest total spent on accessing PHC services at ZAR 459 (28.6 USD) per patient per annum compared to the ‘diabetes-only’ group, which is estimated to have spent ZAR 360 (22.4 USD) per annum. Table 5 shows the percentage of annual income spent—or lost through patient productivity losses—when accessing PHC services in the Agincourt study site. Overall, the costs range from 1.0% to 1.3% of annual income. Although all values are low and not considered CHE as per the definition outlined in the methods. section, individuals with multiple condition incur higher indirect and direct non-health expenses (1.2%) compared to those with a single condition (1.0%) (Table 5).

**Table 5:** Direct non-medical costs and indirect costs, Agincourt study Site, 2022.

	Total sample	HIV-only	DM-only	HT-only	All single conditions (mean)	DM & HT	DM & HIV	HT & HIV	HIV, DM & HT	All MM (mean)
Transport costs per annum (ZAR/USD)	4 867490/303 270	3 065 808/191 016	38 686/2410	899 208/56 025	1 334 567/83 151	203 136/12 656	24 196/1508	589 260/36 714	47 196/2941	215 947/13 455
Patient productivity losses (without caregiver burden) per annum (ZAR/USD)	2 608 340/162 513	1 642 873/102 360	20 731/1292	481 858/30 022	715 154/44 558	108 854/6782	12 966/808	315 767/19 674	25 291/1576	109 397/6816
Total spent on PHC services in Agincourt per patient per annum (ZAR/USD)	373/23.3	367/22.8	360/22.4	365/22.7	364/22.7	436/27.2	459/28.6	398/24.8	450,28.1	411/25.6
Proportion of individual income spent on accessing PHC services (%)	1.1	1.1	1.0	1.0	1.0	1.3	1.3	1.1	1.3	1.2

This highlights the increased societal financial burden on patients managing several chronic illnesses. However, when caregiver costs are included (see Supplementary Appendix S7), the proportion of income spent on healthcare increases from 1.0–1.3% to 14%–18% per annum, constituting CHE for all patient groups with the patients with multiple conditions continuing to face higher CHE. Supplementary Appendix S7 also outlines CHE if literature informed parameters are used which is slightly higher than our estimates.

### Scenario analysis

**Figure 3:**
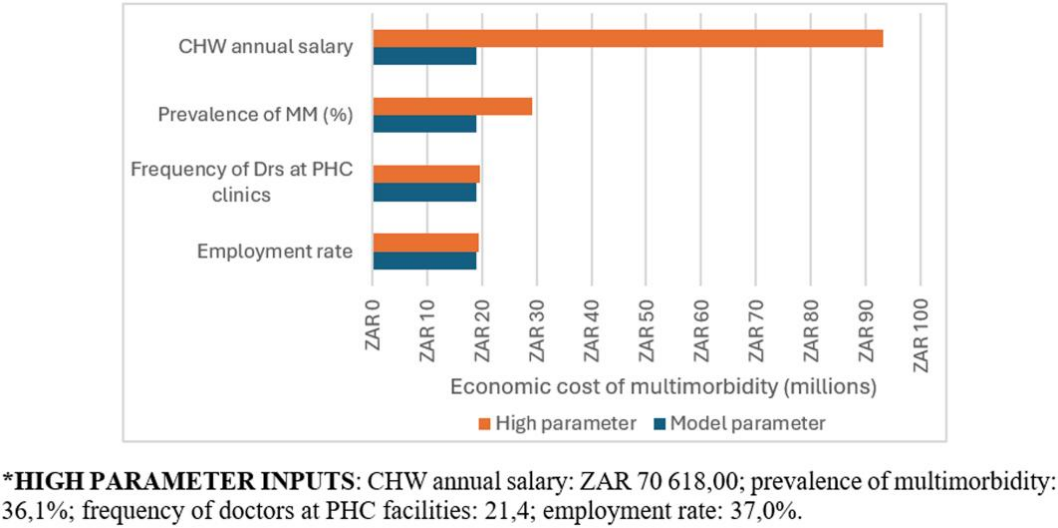
Scenario analyses of the annual economic cost of primary care of multimorbidity.

We assessed scenarios that included an increase in the prevalence of multimorbidity (%), the frequency of doctors’ working at PHC clinics, the annual CHW wage, and the employment rate, as illustrated in Fig. 3. The only scenario which largely increased the annual economic cost of multimorbidity was the CHW annual wages, which was increased by 2% per annum for the next 20 years resulting in nearly five times increased economic cost of managing multimorbidity. This is largely due to the large volume of CHWs who work in the Agincourt study site and the high volume of patients that they see.

## Discussion

This study provides an analysis of the economic burden of outpatient primary care for adults with single and multiple conditions which are highly prevalent in South Africa. This study highlights the financial implications for both the healthcare system and society outlined for each condition group. Overall, the economic cost of treating those with multimorbidity is higher compared to those with single conditions when considering the per patient per annum cost. This is due to higher utilization of healthcare services and increased medication requirements associated with multimorbidity. These findings coincide with findings from global research on the burden and increased costs of multimorbidity (Tran 2024). This study found that patients with two or more conditions had, on average, more annual visits compared to those with a single disease with each additional clinic visit increasing the cost to both the healthcare system and to society. Patients with multimorbidity experience greater indirect costs, predominantly from productivity losses, aligning with global findings that multimorbidity escalates economic burdens due to complex care requirements (Wang et al. 2018, Tran et al. 2022, Tran 2024). This study found that individuals with both HIV and diabetes incurred the highest overall per-patient societal costs of accessing primary healthcare per annum (R459/28.6 USD), highlighting the compounded resource demands of managing chronic non-communicable and infectious diseases concurrently.

It is expected that the resources and costs to manage multimorbidity would exceed those of single conditions, given that each additional condition requires additional condition-specific medication, investigations, and a variety of different healthcare workers. However, we observe in this study that the cost of treating each additional condition is not additive, which may be due to the clinics addressing all conditions in the same sessions in their ‘chronic clinics’. For example, this study estimates that the annual economic cost of primary care for ‘diabetes only’ is R5 637 (351 USD) per patient per annum and ‘hypertension only’ is R4 800 (299 USD) per patient per annum, but the cost of treating a patient with both ‘diabetes and hypertension’ is R6 611 (412 USD) per patient per annum, which is nearly half the cost if one had to add the cost of treating diabetes and hypertension separately (R10 437/649 USD per patient per annum).

The study shows that even though multimorbidity is increasing in prevalence and requires more resources per patient compared to single conditions, offering integrated services where multiple conditions can be treated at the same time, at the same location is likely to result in cost savings. South Africa has already implemented a national-level Integrated Chronic Disease Management (ICDM) model which integrates HIV and NCD care (Matanje Mwagomba et al. 2018). Ameh et al. (2017) assessed the ICDM in its pilot phase in 2011 and found that the ICDM model provided similar health outcomes compared to the standard care offered (single disease model) (Ameh et al. 2017). However, an updated assessment is required to assess the current effects of the ICDM in South Africa.

The findings from this study also highlight the economic vulnerability of individuals with multimorbidity, particularly in rural areas like Agincourt where employment rates are low (Supplementary Appendix S4) and many people, particularly the elderly, rely on grants to survive. Even though healthcare services are free at the point of care in SA, indirect costs such as transportation and productivity losses remain significant barriers which often drive catastrophic health expenditure (CHE) (Mudzengi et al. 2017). Similar trends have been observed in other LMICs. A study in India found that enormous incidence of CHE linked to accessing NCD management with around two-third households with NCDs facing CHE with poorer populations bearing the highest burden, pushing them further into poverty (Lefebvre et al. 2021). A study in SA on HIV and TB had similar findings but showed that patients with both conditions (HIV and TB) faced increased CHE (Mudzengi et al. 2017).

The high level of multimorbidity in South Africa highlights the need for integrated care and a ‘one-stop-shop approach’ where treatment is available, no matter the underlying cause. The need for integrated care has been acknowledged by the Department of Health i.e. integrating HIV, TB, and NCD services with maternal and child health services for efﬁcient services (Roomaney et al. 2021, Roomaney et al. 2023). This was exempliﬁed in the release of the 2016 South African National Department of Health Adherence Guidelines for HIV, TB, and non-communicable diseases (National Department of Health 2021). This policy and the service-delivery guidelines seek to address issues in non-adherence to long-term therapies amidst the expansion of ART programmes and the rising burden of NCDs. Certain aspects of the programme implementation related to this policy have been positively evaluated. Another part of the strategy focused on linkage to care and implementing screening activities to identify diseases early for intervention. While the Adherence Guidelines do not cover every disease combination possible, these are steps in the right direction.

The results underscore a critical challenge for South Africa’s healthcare system: balancing the dual burden of infectious diseases and NCDs. With HIV still highly prevalent and NCDs on the rise, the healthcare system faces a compounded strain (Mayosi et al. 2009, Schneider et al. 2009, Dalal et al. 2011, Lalkhen and Mash 2015). Integrated care models, as demonstrated in this study’s setting, may provide a pathway to mitigate costs—which is further strengthened by research (Tran 2024). However, the successful implementation of integrative services requires significant resource investments in infrastructure, training, and chronic disease management protocols (Tran 2024).

In terms of policy strengthening around multimorbidity, there is a need to strengthen the implementation of cost-effective interventions that integrate the management of NCDs and chronic infectious diseases. For example, task-shifting services to CHWs, like taking vitals on entry to the clinic and at home visits, could alleviate healthcare worker shortages while providing consistent care (Lalkhen and Mash 2015), which we observed in Agincourt. However, we did not have data on the health outcomes of patients which are required to ensure task shifting does not compromise quality care. Second, targeted policies may be required to improve access to PHC services, particularly for patients in rural to reduce indirect costs related to accessing primary healthcare. In this vein, the South African government has various strategies in place to reduce unemployment, such as investing in infrastructure and promoting youth employment programmes (The Presidency of the Republic of South Africa 2025); however, South African unemployment rates continue to be one of the highest in the world. Third, further research is needed to estimate the cost-effectiveness of integrated care models across varying multimorbidity combinations in a variety of contexts (Tran 2024). The heterogeneity of multimorbidity profiles necessitates tailored approaches (Tran 2024, Tran et al. 2024). For example, in this study, hypertension has been shown to be cost limiting, as costs associated with its management are consistently lower compared to diabetes and HIV. In the future, a similar study including a wider variety of conditions would be informative to gauge the prevalence and economic burden of various other prevalent disease combinations in South Africa.

Additional research is required to get further information on the burden of multimorbidity to ensure that future protocols and guidelines for managing multimorbidity in SA are evidence-based and context-specific. Longitudinal studies may be useful to capture the evolving cost burden of disease combinations as these conditions progress beyond the primary care setting along different referral pathways. A cohort simulation model using data from this study and national data could be useful in estimating the cost of multimorbidity across South Africa as a whole.

## Strengths and limitations

### Strengths

- To our knowledge, this is the first COI study on multimorbidity including noncommunicable diseases in South Africa.
- A societal approach was used which estimates the economic burden of multimorbidity instead of just the direct healthcare costs. This gives a better indication of the economic burden of the various conditions.
- The model makes use of a variety of primary and secondary data collected from a rapidly transitioning rural setting (Agincourt), which informs much of the results.

### Limitations

- The definition of multimorbidity in this model does not include all conditions seen in the Agincourt HDSS sub-district and study setting and thus underestimates the full cost of multimorbidity.
- Costs were derived from sources which did not always have the 2022 cost for each item, and so costs had to be inflated to be presented as 2022 cost estimates. Some sources of costs are outdated; for example, the laboratory test costs were from the NHLS 2018 price list, which is the most recent one available.
- Caregiver costs were not considered under indirect costs, but we included them in Supplementary Appendix S7 as based on the literature.
- Health outcomes of patients accessing the clinics in Agincourt were not investigated and so quality of care has not been assessed. Much of the healthcare service provided for the conditions in this study are managed by CHWs and nurses in the chronic clinics. Ensuring good health outcomes from integrated care clinics is crucial when advocating for integration of care in future.
- The costs associated with HCW overtime have not been included and thus may underestimate the true cost of managing patients in primary care settings. Only costs associated with public primary care are included in the model. This neglects the costs related to the complications that occur in each condition, for example, a stroke resulting from poorly managed hypertension.

## Conclusion

A consequence of rapid health and social transitions, increasing prevalence of NCDs and multimorbidity creates a complex healthcare issue globally and in South Africa. This study found that multimorbidity is more costly to treat per patient than those with single diseases; however, if patients with multimorbidity are treated in an integrated care setting, the economic burden of multiple conditions can be contained. Integrated care seems to offer a way to treat patients with multimorbidity in a cost-limiting manner. However, further investigation is needed to ensure that integrated care in South Africa leads to good health outcomes. Guidelines and protocols for treating patients with multimorbidity are not yet available in SA, and further research is needed to develop and implement these to ensure efficient, quality, and cost-effective multimorbidity management. Policies should focus on strengthening the existing integrated care mechanisms in SA, improving access to PHC services by patients with multimorbidity and decreasing the societal burden of individuals with multimorbidity. Further research on multimorbidity with a focus on NCDs will be essential if South Africa is to make progress in its efforts to implement universal healthcare.

## Supplementary Material

czag016_Supplementary_Data

## Data Availability

The patient-level data used for this study is known as ‘Clink Link’ data and is the property of Agincourt HDSS (South African Population Research Infrastructure Network (SAPRIN) 2025). To access this data, please contact the owners of the data: The MRC/Wits Rural Public Health and Health Transitions Unit based at the University of Witwatersrand (https://www.agincourt.co.za/contact-info-2).

## Reflexivity statement

The authors of this study represent a diverse team with varied genders, ethnicities, ages, backgrounds, and levels of professional experience. Collectively, we bring a variety of expertise spanning public health, rural health, data science, health economics, bioinformatics, biostatistics, and clinical practice within the South African context. The first author is an early-career researcher, and the development of this paper has contributed to ongoing capacity building in the field of health economics in South Africa.
