# Supplementary material for: The economic cost of outpatient primary care of adults with multimorbidity (HIV, diabetes, and hypertension) in rural South Africa
Source: Health Policy Plan. 2026 Feb 10;41(4):570–83. doi: 10.1093/heapol/czag016 (PMC13089540; doi:10.1093/heapol/czag016)
Supplement: czag016_Supplementary_Data [file czag016_supplementary_data.zip › APPENDIX 1.docx]

**APPENDIX 1:** Clinic Link data variables: Medication and laboratory test datasets

*All data in the tables below are made up for illustrative purposes only.*

**Table A:** Clinic Link data variables: Medication dataset

| Patient ID | Date | Diagnosis 1 | Diagnosis 2 | Diagnosis 3 | Treatment 1 | Amount Of Med Given 1 | Daily Dosage 1 |
| --- | --- | --- | --- | --- | --- | --- | --- |
| 1001 | 01-01-2022 | HIV | TB | Hypertension | Metformin | 56 | 2 |
| 1002 | 02-05-2022 | Hypertension |  |  | Simvastatin | 56 | 1 |
| 1002 | 08-10-2022 | Hypertension |  |  | Panado | 20 | 3 |
| 1004 | 24-02-2022 | Diabetes | Hypertension |  | Insulin | 10 |  |

**Table B:** Clinic Link data variables: Laboratory tests dataset

| **PatientId** | **Lab_test_name** | **Date** |
| --- | --- | --- |
| 1001 | CREATININE(EGFR) | 31-01-22 |
| 1002 | VIRALLOAD | 07-09-22 |
| 1003 | CREATININE(EGFR) | 07-09-22 |
