# Supplementary material for: The economic cost of outpatient primary care of adults with multimorbidity (HIV, diabetes, and hypertension) in rural South Africa
Source: Health Policy Plan. 2026 Feb 10;41(4):570–83. doi: 10.1093/heapol/czag016 (PMC13089540; doi:10.1093/heapol/czag016)
Supplement: czag016_Supplementary_Data [file czag016_supplementary_data.zip › Appendix 10.docx]

**Appendix 10:** Clinic Link Laboratory test grouping

| **Laboratory test variable name from the Clinic Link dataset** | **Name of laboratory test in NHLS most suited** |
| --- | --- |
| ALBUMIN | Albumin |
| BLOOD COUNT | Full Blood Count |
| CD4%_OF_LYMPHOCYTES | CD4 Helper T Cell Marker |
| CD45_+VE_CELL_COUNT | CD4 Helper T Cell Marker |
| CD4COUNT | CD4 Helper T Cell Marker |
| CERVICAL SMEAR | EXF Cytology (Gynae) 1st Smear |
| CERVICAL SMEAR (CERVIX) | EXF Cytology (Gynae) 1st Smear |
| CHOLESTEROL | Cholesterol Total |
| CREATININE(EGFR) | Creatinine |
| CRYPTOCOCCAL_ANTIGEN_TEST(CRAG) | Cryptococcal Antigen - Titre |
| GENEPERT | GeneXpert PCR TB |
| GENEXPERT | GeneXpert PCR TB |
| GLUCOSE | Glucose |
| GLUCOSE (FASTING) | Glucose |
| HAEMOGLOBIN | Hb Only |
| HBA1C | Glycated Haemoglobin |
| HEPATITIS B CORE IGM | Hepatitis B Core IgM |
| HEPATITIS B E AG | Hepatitis B E Ag |
| HEPATITIS_A_LGM | Hepatitis A IgG |
| HEPATITIS_B_SURFACE_AG | Hepatitis B Surface Ag |
| LDL_CHOLESTEROL | Cholesterol LDL - Measured |
| LEUCOCYTES | WBC Only |
| M.TUBERCULOSIS | PCR for M. tuberculosis |
| POTASSIUM | Potassium |
| RHESU FACTOR (D) | Rh Only |
| RPR (RAPID PLASMA REAGIN) | RPR Screen |
| RPR SCREEN | RPR Screen |
| SODIUM | Sodium |
| SURFACE ANTIGEN RESULT (Heb B) | Hepatitis B Surface Ag Rapid **or**  Hepatitis B Surface Ag **or**  Hepatitis B Surface Ag Confirm  **or**  Hepatitis B Surface Ab **or**  Hepatitis B Surface Ag Neut |
| SYPHILIS | Treponema Pallidum ab Test |
| TB CULTERE | R/M Culture TB |
| TB_GENEXPERT | GeneXpert PCR TB |
| TB_MICROSOPY | Fluid Prep For TB Microscopy and  TB Microscopy Auramine |
| TOTAL CHOLESTEROL | Cholesterol Total |
| TOTAL PROTEIN | Protein Total |
| TOTAL_BILLIRUBIN | Bilirubin Total |
| TRGLYCERIDES | Triglyceride |
| UREA | Urea |
| URIC_ACID | Uric Acid |
| VIRALLOAD | HIV Viral Load |
