# Supplementary material for: The economic cost of outpatient primary care of adults with multimorbidity (HIV, diabetes, and hypertension) in rural South Africa
Source: Health Policy Plan. 2026 Feb 10;41(4):570–83. doi: 10.1093/heapol/czag016 (PMC13089540; doi:10.1093/heapol/czag016)

**APPENDIX 2:** Indirect Costs Questionnaire used to collect data on travel costs and productivity losses in Agincourt


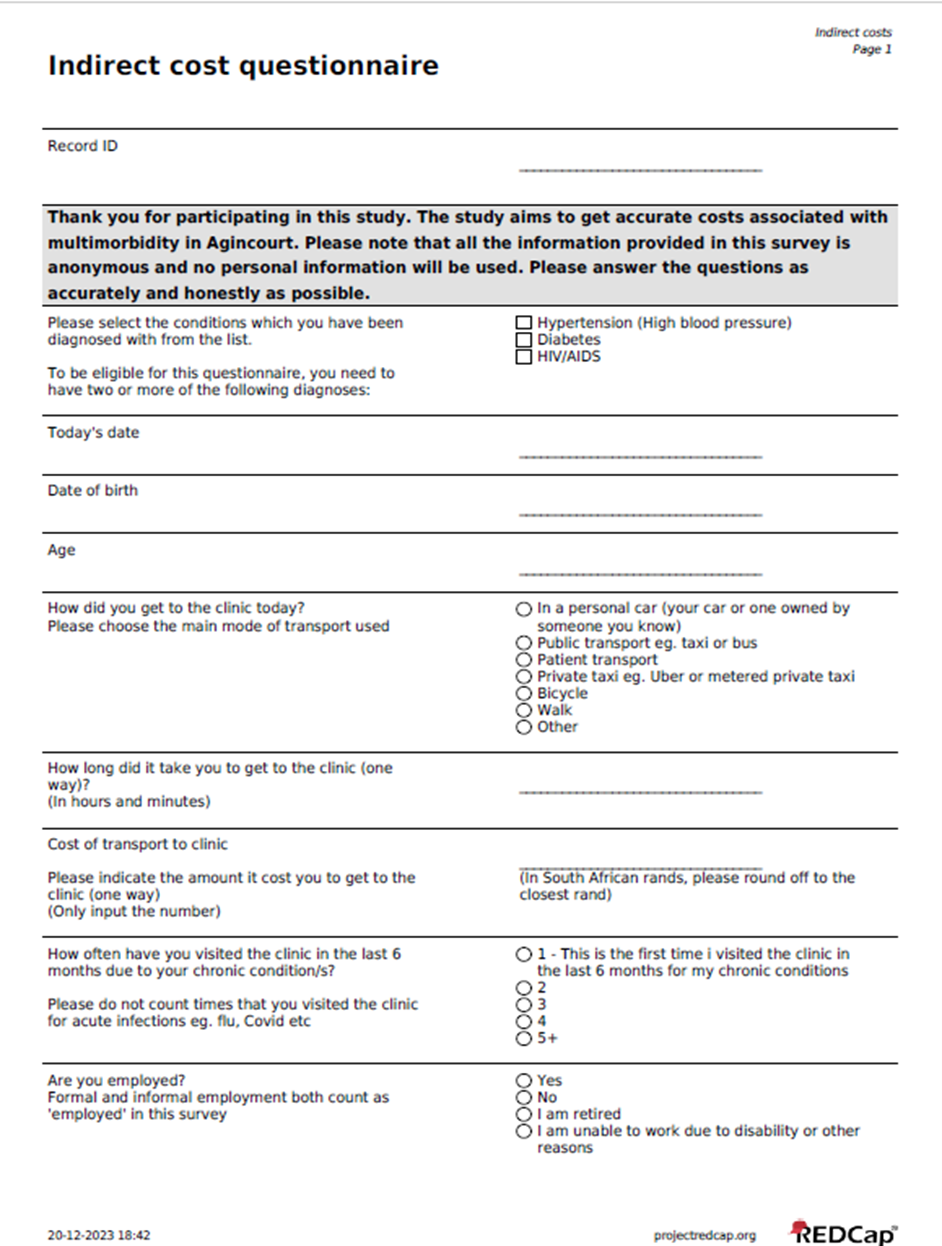


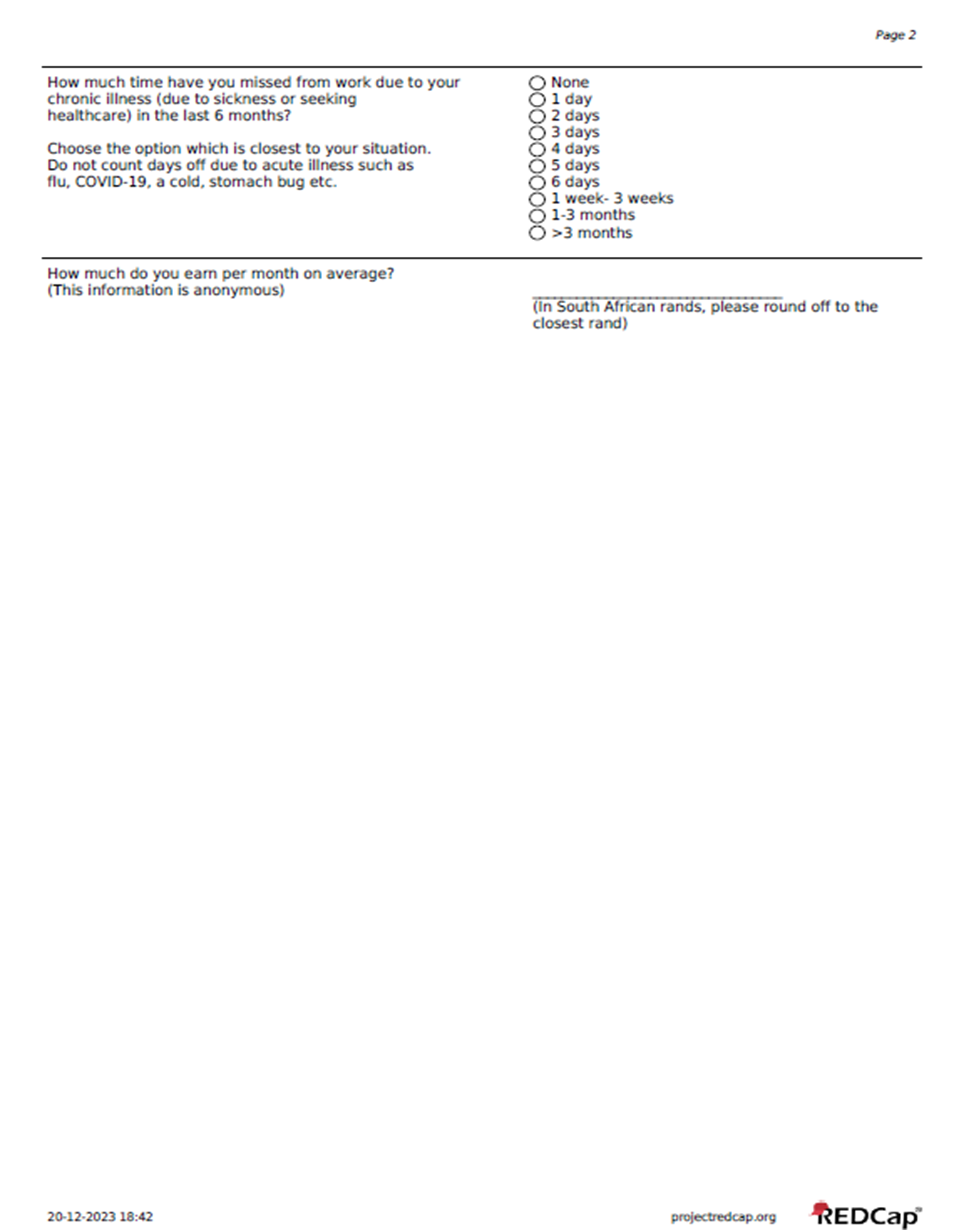

Supplement: czag016_Supplementary_Data [file czag016_supplementary_data.zip › APPENDIX 2.docx]
