# Supplementary material for: The economic cost of outpatient primary care of adults with multimorbidity (HIV, diabetes, and hypertension) in rural South Africa
Source: Health Policy Plan. 2026 Feb 10;41(4):570–83. doi: 10.1093/heapol/czag016 (PMC13089540; doi:10.1093/heapol/czag016)

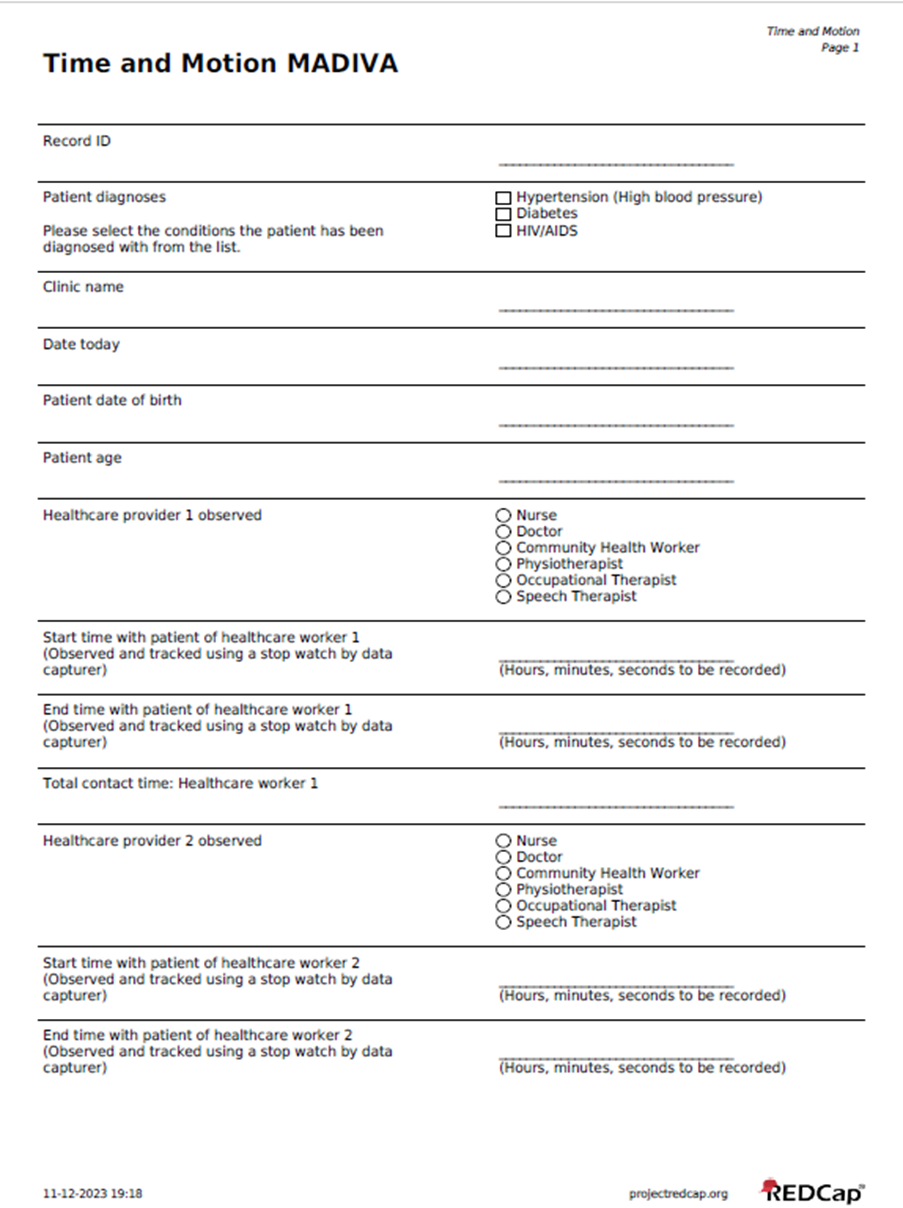
**APPENDIX 3:** Time and Motion data collection tool to glean healthcare worker time spent with patients


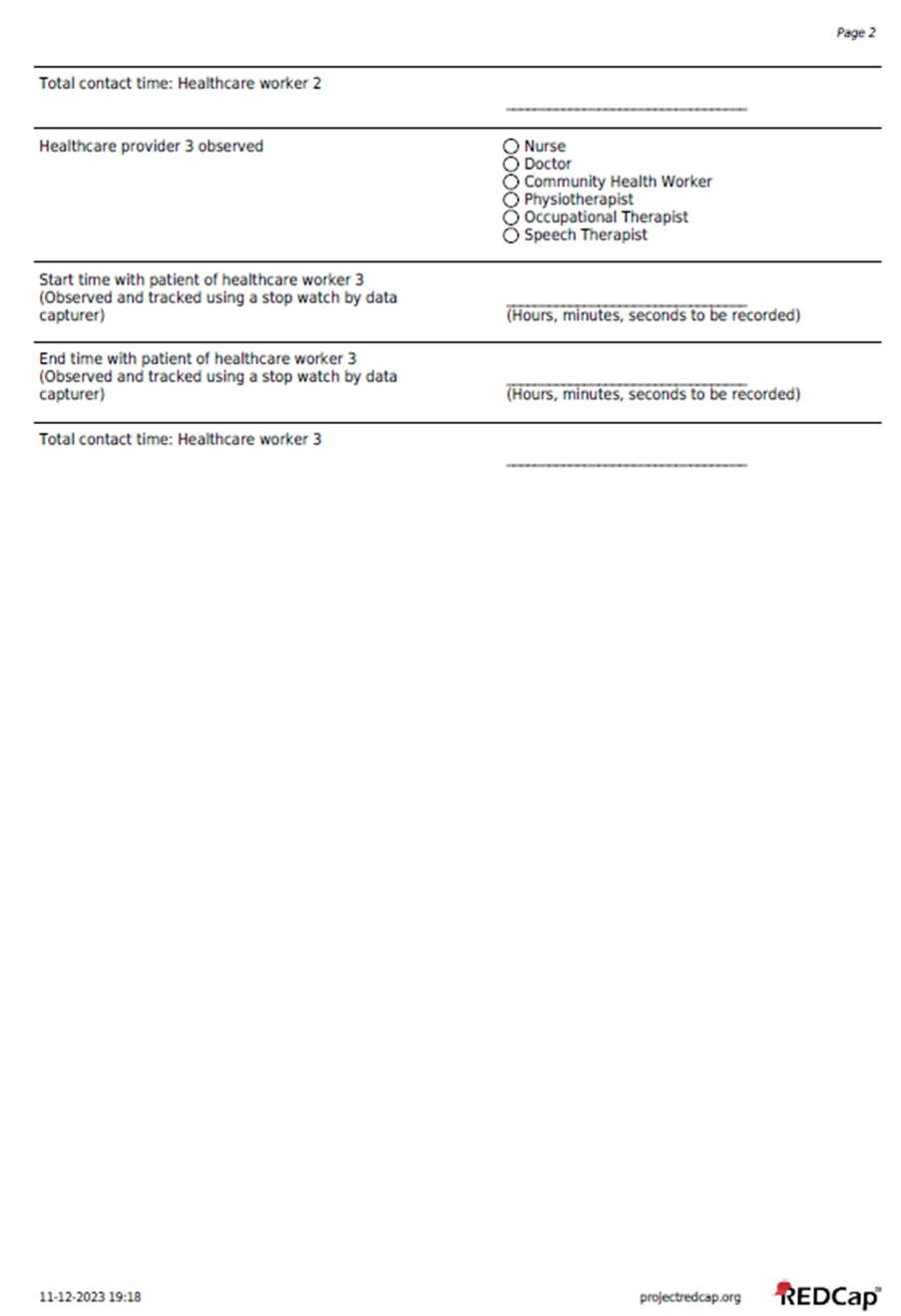

Supplement: czag016_Supplementary_Data [file czag016_supplementary_data.zip › APPENDIX 3.docx]
