# Supplementary material for: The economic cost of outpatient primary care of adults with multimorbidity (HIV, diabetes, and hypertension) in rural South Africa
Source: Health Policy Plan. 2026 Feb 10;41(4):570–83. doi: 10.1093/heapol/czag016 (PMC13089540; doi:10.1093/heapol/czag016)
Supplement: czag016_Supplementary_Data [file czag016_supplementary_data.zip › APPENDIX 4.docx]

**APPENDIX 4:** Indirect Cost sub-study results - summarised

Summary of sub-study: There is very limited data on the indirect costs and the time various healthcare workers spend with patients, particularly those associated with accessing public PHC services in SA in rural areas like Agincourt. Thus, we undertook a sub-study where we collected primary data from 3 PHC facilities in Agincourt (Agincourt CHC, Lillydale clinic and Kildare clinic) to get estimates of the indirect costs and productivity losses associated with accessing PHC services in Agincourt. The mean and median age of the sample was 49 years old. 28% of the sample was 60 years or older, which is quite a lot higher than the STATS SA estimation of the proportion of 60+ population at 8,2% in MP in 2022 (Statistics South Africa (Stats SA), 2022b).

Indirect cost study sample: In the sample (n=47), 57% had hypertension, 55% had HIV, and 21% had DM. Most of the sample (35 patients) had just one of the conditions and only 12 patients in the sample had multimorbidity. As there are so few patients with multimorbidity, the average results across all the patients used in this study to determine the indirect costs associated with accessing PHC services in Agincourt.

Summary of results:

| **Indirect Cost Results** | | |
| --- | --- | --- |
| **Variable** | **Unit of result** | **Results** |
| Cost of transport to clinic (both ways) in rands | Mean (min; max) | R46,00 (0,00-R600,00) |
| Visits per year for chronic illness | Mean (min; max) | 4 (1; 5+) |
| Employment rate of sample | Total percentage | 17% |
| Income of sample (median) | Median | R2900 |
| Income of sample | Median | R170,00 |
