# Supplementary material for: The economic cost of outpatient primary care of adults with multimorbidity (HIV, diabetes, and hypertension) in rural South Africa
Source: Health Policy Plan. 2026 Feb 10;41(4):570–83. doi: 10.1093/heapol/czag016 (PMC13089540; doi:10.1093/heapol/czag016)
Supplement: czag016_Supplementary_Data [file czag016_supplementary_data.zip › APPENDIX 5.docx]

**APPENDIX 5:** Time and Motion sub-study results - summarised

Summary of the sub-study: There is very limited data on the indirect costs and the time various healthcare workers spend with patients, particularly those associated with accessing public PHC services in SA in rural areas like Agincourt. Thus, we undertook a sub-study where we collected primary data from 3 PHC facilities in Agincourt (Agincourt CHC, Lillydale clinic and Kildare clinic) to get estimates of the indirect costs and productivity losses associated with accessing PHC services in Agincourt.

Study sample: A total of 34 sessions observed which included 30 patients, most of which either saw a nurse or CHW except for two patients who saw both in one day. Of the 30 patients observed, 20 patients in the sample had a single condition and only 10 had multimorbidity. In terms of HCWs, 14 sessions were with CHWs and 20 were with nurses. No doctors were present at any of the clinics we collected data from during the 5-day data collection period. The mean and median age of the sample was 49 years old. 28% of the sample was 60 years or older, which is quite a lot higher than the STATS SA estimation of the proportion of 60+ population at 8,2% in MP in 2022 (Statistics South Africa (Stats SA), 2022b).

Summary of results:

| **Time and Motion results** | |
| --- | --- |
| **Time spent by nurses in one session (mean)** | |
| All patients | 10 min, 49 secs |
| Single condition patients | 8 min, 12 secs |
| MM patients | 15 min, 10 secs |
| Ratio (MM patients/single condition patients) | 1,85 |
| **Time spent by CHWs in one session (mean)** | |
| All patients | 7 min, 0 secs |
| Single condition patients | 5 min, 0 secs |
| MM patients | 10 min, 9 secs |
| Ratio (MM patients/single condition patients) | 2,03 |
| **Time spent by nurses and CHWs in one session (mean)** | |
| All patients | 8min, 2 secs |
| Single condition patients | 6min, 20 secs |
| MM patients | 11min, 30 secs |
| Ratio (MM patients/single condition patients) | 1,82 |
