# Supplementary material for: The economic cost of outpatient primary care of adults with multimorbidity (HIV, diabetes, and hypertension) in rural South Africa
Source: Health Policy Plan. 2026 Feb 10;41(4):570–83. doi: 10.1093/heapol/czag016 (PMC13089540; doi:10.1093/heapol/czag016)
Supplement: czag016_Supplementary_Data [file czag016_supplementary_data.zip › APPENDIX 6.docx]

**APPENDIX 6:** Healthcare workers across the 8 PHC clinics in Agincourt, according to data collectors from WITS Agincourt

| **Healthcare Worker type** | **Total number across 8 PHC clinics** | **Total number across 8 PHC clinics based on their frequency at each clinic** |
| --- | --- | --- |
| Operations Manager G1 | 8 | 8 |
| Professional nurse G1 | 110 | 110 |
| Community Health workers | 154 | 154 |
| Medical Officer (doctor) G1 | 6 | 1,4 |
| Physio G1 | 5 | 0,25 |
| OT G1 | 3 | 0,15 |
| Speech G1 | 4 | 0,2 |
| Audiologist G1 | 4 | 1,4 |
| Pharmacist G1 | 8 | 8 |
| Pharmacist assistant | 8 | 8 |
| HIV lay counsellor | 6 | 6 |
| Dietician G1 | 6 | 0,3 |
| Optometrist G1 | 2 | 0,6 |
| Psychologist G1 | 4 | 0,2 |
