# Supplementary material for: The economic cost of outpatient primary care of adults with multimorbidity (HIV, diabetes, and hypertension) in rural South Africa
Source: Health Policy Plan. 2026 Feb 10;41(4):570–83. doi: 10.1093/heapol/czag016 (PMC13089540; doi:10.1093/heapol/czag016)
Supplement: czag016_Supplementary_Data [file czag016_supplementary_data.zip › APPENDIX 7.docx]

**APPENDIX 7:** Indirect costs associated with accessing Primary Healthcare in Agincourt using literature-based figures compared to the primary data collected in Agincourt

|  |  | **TOTAL SAMPLE** | **HIV ONLY** | **DM ONLY** | **HT ONLY** | **DM & HT** | **DM & HIV** | **HT & HIV** | **HIV, DM & HT** | **TOTAL ALL MM** |
| --- | --- | --- | --- | --- | --- | --- | --- | --- | --- | --- |
| **Transport costs** | Total cost per annum | R4 867 490 | R3 065 808 | R38 686 | R899 208 | R203 136 | R24 196 | R589 260 | R47 196 | R816 592 |
|  | Cost per patient per annum | R243 | R239 | R234 | R238 | R284 | R299 | R259 | R293 | R266 |
| **Patient productivity losses - *Literature informed*** | Total cost per annum | R11 418 497 | R7 191 986 | R90 752 | R2 109 425 | R476 531 | R56 761 | R1 382 327 | R110 716 | R1 915 618 |
|  | Cost per patient per annum | R570 | R560 | R550 | R557 | R666 | R701 | R608 | R688 | R624 |
| **Patient productivity losses - *Agincourt sub-study*** | Total Cost per annum | R2 608 340 | R1 642 873 | R20 731 | R481 858 | R108 854 | R12 966 | R315 767 | R25 291 | R437 587 |
|  | Cost per patient per annum | R130 | R128 | R126 | R127 | R152 | R160 | R139 | R157 | R143 |
| **Caregiver productivity losses - *Literature informed*** | Total cost per annum | R157 135 275 | R98 972 280 | R1 248 885 | R29 028 780 | R6 557 760 | R781 110 | R19 022 850 | R1 523 610 | R26 361 720 |
|  | Cost per patient per annum | R7 847 | R7 706 | R7 569 | R7 669 | R9 172 | R9 643 | R8 365 | R9 463 | R8 587 |
| **Caregiver productivity losses - *Agincourt sub-study*** | Total cost per annum | R92 059 050 | R57 983 760 | R731 670 | R17 006 760 | R3 841 920 | R457 620 | R11 144 700 | R892 620 | R15 444 240 |
|  | Cost per patient per annum | R4 597 | R4 514 | R4 434 | R4 493 | R5 373 | R5 650 | R4 901 | R5 544 | R5 031 |
| **Total spent on PHC services in Agincourt per patient per annum** (Agincourt sub-study) | | R5 541 | R5 441 | R5 344 | R5 415 | R6 476 | R6 809 | R5 907 | R6 682 | R6 063 |
| **Percentage of annual income spent on accessing PHC services in Agincourt** (Agincourt sub-study) | | 15,9% | 15,6% | 15,4% | 15,6% | 18,6% | 19,6% | 17,0% | 19,2% | 17,4% |
| **Total spent on PHC services in Agincourt per annum** - (Literature) | | R8 660 | R8 504 | R8 353 | R8 464 | R10 122 | R10 643 | R9 232 | R10 444 | R9 477 |
| **Percentage of annual income spent on accessing PHC services in Agincourt** (Literature) | | 14,6% | 14,3% | 14,1% | 14,2% | 17,0% | 17,9% | 15,5% | 17,6% | 16,0% |

**Appendix 7 notes:**

The Agincourt sub study found an employment rate of 17% which is quite low compared to the published unemployment rate in MP in 2022 as 44% when accounting for the percentage of people over 60 in MP (8,2%)(Statistics South Africa (Stats SA), 2022b). It is possible that our sample had a much lower employment rate as they situated in a rural area of MP. Another reason may be that the sample we collected data from included 3 times more people over 60 (28%) compared to the 8,2% estimate of people over 60 published by STATS SA for MP in 2022 (Table 5).

We did not have data on caregivers from our Agincourt sample but in this table we used the results taken from a South African COI paper on TB and HIV (Mudzengi *et al.*, 2017). Their sample comprised of HIV and TB patients seeking primary care in Ekurhuleni North Sub-District, SA and found that 55% of HIV patients had informal caregivers, 37% of TB patients had carers and patients with both TB and HIV had 48% of carers. In the absence of data collection in this area we have considered the results from the study above and have taken a conservative approach, assuming that 30% of all patients in our sample have informal carers. The median salary for MP in 2022 was used to calculate the productivity losses for the informal caregivers (Statistics South Africa (Stats SA), 2024b).
