# Supplementary material for: The economic cost of outpatient primary care of adults with multimorbidity (HIV, diabetes, and hypertension) in rural South Africa
Source: Health Policy Plan. 2026 Feb 10;41(4):570–83. doi: 10.1093/heapol/czag016 (PMC13089540; doi:10.1093/heapol/czag016)
Supplement: czag016_Supplementary_Data [file czag016_supplementary_data.zip › APPENDIX 8.docx]

**APPENDIX 8:** Accounting for missing values and incorrect values in the 2022 Clinic Link medication dataset

- Missing values replaced with the most reported value for that medication according to the Clinic Link dataset
- Missing values are replaced with values >1 as we assumed an “intention to treat” meaning that if the medication was mentioned in the patient’s visit in the Clinic Link dataset we assumed that the health professional prescribed the medication to that patient even if no (zero) units were recorded. In many cases, no units were recorded alongside the medication name and If we kept all missing values as ‘missing’ or zero, this would grossly underestimate the amount and cost of medication required to treat each patient group.

| **Variable name** | **Change made** | **Condition** |
| --- | --- | --- |
| 3TC/AZT | Change all missing values to ‘56’ | HIV |
| 3TC | Change all missing values to ‘56’ | HIV |
| 3TC | Change all missing values to ‘56’ | HIV |
| ABACAVIR | Change all missing values to ‘56’ | HIV |
| ABC/3TC | Change all missing values to ‘56’ | HIV |
| ABC | Change all missing values to ‘56’ | HIV |
| ACTRAPHANE | Change all missing values to ‘5’ and change ‘112’ to ‘5’; change ‘56’ to ‘5’; change ‘28’ to ‘5’ | DIABETES MELLITUS |
| ACTRAPID | Change all missing values to ‘5’and change ‘104’ to ‘5’ and change ‘104’ to ‘5’ | DIABETES MELLITUS |
| ADALAT | Change all missing values to ‘56’ | HYPERTENSION |
| ALDACTONE | Change all missing values to ‘28’ | HYPERTENSION |
| ALDOMET | Change all missing values to ‘56’ | HYPERTENSION |
| ALUVIA | Change all missing values to ‘84’ | HIV |
| AMLOC | Change all missing values to ‘56’ | HYPERTENSION |
| AMLODIPINE | Change all missing values to ‘56’ | HYPERTENSION |
| ATAZANAVIR | Change all missing values to ‘28’ | HIV |
| ATENOLOL | Change all missing values to ‘56’ | HYPERTENSION |
| AZT | Change all missing values to ‘56’ | HIV |
| CALCIUM GLUCONATE | Change all missing values to ‘28’ | HYPERTENSION |
| CARDURA | Change all missing values to ‘56’ | HYPERTENSION |
| CARLOC CARVEDILOL | Change all missing values to ‘56’ | HYPERTENSION |
| CARLOC | Change all missing values to ‘28’ | HYPERTENSION |
| CARLOC-CARVEDILOL | Change all missing values to ‘56’ | HYPERTENSION |
| CARVEDILOL | Change all missing values to ‘28’ | HYPERTENSION |
| COVERSYL | Change all missing values to ‘56’ | HYPERTENSION |
| DAONIL | Change all missing values to ‘28’ | DIABETES MELLITUS |
| DAPAMAX | Change all missing values to ‘28’ | DIABETES MELLITUS |
| DIGOXIN | Change all missing values to ‘56’ | HYPERTENSION / HEART FAILURE |
| DOLUTEGRAVIR | Change all missing values to ‘56’ | HIV |
| DTG | Change all missing values to ‘28’ | HIV |
| DUMIVA | Change all missing values to ‘56’ | HIV |
| EFAVIRENZ | Change all missing values to ‘56’ | HIV |
| EFV | Change all missing values to ‘56’ | HIV |
| EMTRICITABINE | Change all missing values to ‘56’ | HIV |
| ENALAPRIL | Change all missing values to ‘56’ | HYPERTENSION |
| FDC | Change all missing values to ‘56’ | HIV |
| FEROSEMIDE | Change all missing values to ‘56’ | HYPERTENSION / HEART FAILURE / OEDEMA |
| FTC | Change all missing values to ‘28’ | HIV |
| GLIBENCLAMIDE | Change all missing values to ‘56’ | DIABETES MELLITUS |
| GLICLAZIDE | Change all missing values to ‘28’ | DIABETES MELLITUS |
| GLUCOPHAGE | Change all missing values to ‘112’ | DIABETES MELLITUS |
| GLYCOMIN | Change all missing values to ‘28’ | DIABETES MELLITUS |
| HCTZ | Change all missing values to ‘56’ | HYPERTENSION |
| HYDROCHLOROTHIAZIDE | Change all missing values to ‘56’ and ‘1’ to ‘28’ and ‘2’ to ‘56’ | HYPERTENSION |
| INDAPIMIDE | Change all missing values to ‘28’ | HYPERTENSION |
| INSULIN: (DM) | Change all missing values to ‘2’ and change ‘1000’ to ‘2’ and ‘28’ to ‘2’ | DIABETES MELLITUS |
| IPT | Change all missing values to ‘56’ | TB/HIV |
| IPT | Change all missing values to ‘56’ | TB/HIV |
| ISOSORBIDE | Change all missing values to ‘56’ | CVD |
| LAMIVUDINE | Change all missing values to ‘28’ | HIV |
| LASIX | Change all missing values to ‘56’ | HYPERTENSION/OEDEMA |
| LTD | Change all missing values to ‘56’ | HIV |
| METFORMIN | Change all missing values to ‘84’ | HYPERTENSION |
| METHYLDOPA | Change all missing values to ‘84’ | HYPERTENSION |
| METRORMIN | Change all missing values to ‘84’ | DIABETES MELLITUS |
| NIFEDIPINE | Change all missing values to ‘30’ | HYPERTENSION |
| NVP | Change all missing values to ‘56’ | HIV |
| PERINDOPRIL | Change all missing values to ‘28’ | HYPERTENSION |
| PREXUM PLUS | Change all missing values to ‘56’ | HYPERTENSION |
| PREXUM | Change all missing values to ‘5’ | HYPERTENSION |
| PROTAPHANE: | Change all missing values to ‘5’ | DIABETES MELLITUS |
| RIDAQ | Change all missing values to ‘28’ | HYPERTENSION/OEDEMA |
| RITONAVIR | Change all missing values to ‘30’ | HIV |
| ROTANAVIR | Change all missing values to ‘30’ | HIV |
| SIMVASTATIN | Change all missing values to ‘56’ | CVD |
| SPIRANACTONE | Change all missing values to ‘56’ | HYPERTENSION |
| SPIRONOLACTONE | Change all missing values to ‘56’ | HYPERTENSION/HEART FAILURE |
| STOCRIN | Change all missing values to ‘30’ | HIV |
| TDF/FTC | Change all missing values to ‘28’ | HIV |
| TDF | Change all missing values to ‘28’ | HIV |
| TEE | Change all missing values to ‘56’ | HIV |
| TENEMINE | Change all missing values to ‘56’ | HIV |
| TENOFOVIR | Change all missing values to ‘28’ | HIV |
| TLD | Change all missing values to ‘56’ | HIV |
| TRUVADA | Change all missing values to ‘56’ | HIV |
| ZIDOVUDINE | Change all missing values to ‘60’ | HIV |
| ZINPLEX | Change all missing values to ‘28’ | HYPERTENSION |
| ZOCOR | Change all missing values to ‘56’ | CVD |
| ZOCOR | Change all missing values to ‘56’ | CVD |
| ZOVILAM | Change all missing values to ‘56’ | HIV |
