# Supplementary material for: The economic cost of outpatient primary care of adults with multimorbidity (HIV, diabetes, and hypertension) in rural South Africa
Source: Health Policy Plan. 2026 Feb 10;41(4):570–83. doi: 10.1093/heapol/czag016 (PMC13089540; doi:10.1093/heapol/czag016)
Supplement: czag016_Supplementary_Data [file czag016_supplementary_data.zip › Appendix 9.docx]

**Appendix 9:** Clinic Link Medication grouping

| **Medication name in model** | **Variable names in Clinic Link dataset** |
| --- | --- |
| 3HP | v4 |
| 3TC/TDF | tctdf |
| Abacavir | abacavir  abc |
| Actraphane – insulin | actraphane  insulin  v254 |
| Actrapid – insulin | actrapid |
| Adalat – Nifedipine | adalat  nifedipine |
| Aldactone – Simvastatin | aldactone  simvastatin |
| Aldactone – Spironolactone | aldactone  spironolactone  spiranactone |
| Aluvia - Lopinavir and Ritonavir | aluvia  lopinavir |
| Amloc – Amlodipine | amloc  amlodipine |
| Atazanavir | atazanavir  azt |
| Atazanavir and Lamivudine | azt3tc |
| Atenolol | atenolol |
| Calcium gluconate/calcium salt/zinplex | calciumgluconate  calciumglucose |
| Cardura – Doxizosin | cardura |
| Carloc – Carvedilol | carloccarvedilol  carvedilol |
| Daonil | daonil  dionil |
| Dapamax | dapamax |
| Digoxin | digoxin |
| DTG - Dolutegravir | dolutegravir  dtg |
| Dumiva – Abacavir and Lamivudine (3TC and ABC) | dumiva  tcabc  abc3tc |
| EFV – Efavirenz | efavirenz  efv  v169 |
| Enalapril | enalapril  enapril |
| FDC – Fixed dose combination (TLD) | fdc  v185  tld  v443  ltd |
| FTC - Emtriciabine | ftc  emtricitabine |
| Glibenclamide | glibenclamide |
| Gliclazide | gliclazide |
| Glimepride | glimepiride |
| Glycomin | glycomin |
| Insulin | insulin |
| IPT: Isoniazid preventative therapy | ipt  V257  isoniazid |
| Lamivudine | tc  lamivudine |
| Lasix | lasix |
| Metformin | metformin |
| Methyldopa | methyldopa |
| Methyldopa - Aldomet | aldomet |
| Nifedipine | nifedipine |
| NVP: Nevirapine | nevirapine  nvp |
| Perindopril | Perindopril  perindoprilplus |
| Pharmapress: Enalapril and HCTZ | pharmapress |
| Phenerine | phenerine |
| Prexum | prexum  prexumplus  V375 |
| Propranol | propranolol |
| Protaphane | protaphane |
| Ridaq – Hydrochlorothiazide (HCTZ) | ridaq  hctz  hydrochlorothiazide  indapamide |
| Ritonavir | ritonavir  lopinavir |
| Ritonavir | ritonavir |
| Simvastatin | simvastatin |
| TDF/FTC | tdfftc |
| TEE | tee |
| Tenofovir | tdf  tenofovir  tdf/ftc |
| Truvada | truvada  tenemine  tdfftc |
| Zidovudine | azt/zidovdine |
| Zocor - Simvastatin | zocor  zozor |
| Zovilam – 3TC and AZT | tcazt  azt3tc |
